# Exploration of acupuncture therapy in the treatment of mild cognitive impairment based on the brain-gut axis theory

**DOI:** 10.3389/fnhum.2022.891411

**Published:** 2022-09-20

**Authors:** Yuanyuan Jin, Fen Hu, Jianfang Zhu

**Affiliations:** ^1^Department of Acupuncture and Moxibustion, Zhejiang Hospital, Hangzhou, China; ^2^Department of Acupuncture and Moxibustion, Zhejiang Chinese Medical University, Hangzhou, China

**Keywords:** mild cognitive impairment, acupuncture, gut microbiota, mechanism research, randomized controlled trail

## Abstract

**Background:**

Mild cognitive impairment (MCI) is an intermediary state between normal aging and dementia. Early intervention for MCI may be a key opportunity in managing dementia. Recent studies have demonstrated the alterations in the gut microbial communities associated with MCI. This study aims to evaluate if acupuncture can improve cognitive function in subjects with MCI and explore the possible mechanism of acupuncture by better defining the interactions of gut microbiota.

**Methods:**

A randomized assessor-blind controlled study is proposed. A total of 62 subjects will be recruited and randomly allocated into two groups in a 1:1 ratio: the treatment and control groups. Participants in the treatment group will receive active acupuncture and exercise/cognitive training (conventional treatment). The control group will receive sham acupuncture and exercise/cognitive training. Each participant will receive active or sham acupuncture for 12 weeks. The primary outcome will be the Montreal Cognitive Assessment (MoCA) score and intestinal flora. Secondary outcomes will include mini-mental state examination (MMSE) and activity of daily living (ADL) scores. Various scales will be collected at baseline, during the treatment (weeks 4 and 8), week 12, and months 4 and 6 after the intervention. Feces will be collected before and after the treatment based on 16S rRNA gene sequencing technology for each participant to characterize the intestinal flora. Adverse events will be recorded by monthly follow-up.

**Results:**

The trial is expected to show that cognitive function can be improved by acupuncture and produce reliable clinical outcomes in MCI patients. It will also provide preliminary data on the possible mechanism based on the changes in the intestinal flora. Collected data will be used to support future large-scale fundamental studies.

**Conclusion:**

Acupuncture is an effective method to improve cognitive function for MCI. This study will provide data on the relationship between gut microbiota and the effectiveness of acupuncture in patients with MCI from a new angle.

**Clinical trial registration:**

[www.ClinicalTrials.gov], identifier [MR-33-22-002376].

## Introduction

Mild cognitive impairment (MCI) refers to the progressive decline in memory or other cognitive functions that neither affects the activities of daily life nor meets the diagnostic criteria of dementia (Petersen, 2011). MCI is a clinical state between Alzheimer’s disease (AD) and normal aging. The prevalence of MCI increases with age: 6.7% at 60–64 years, 8.4% at 65–69 years, 10.1% at 70–74 years, 14.8% at 75–79 years, and 25.2% at 80–84 years (Petersen et al., 2017). In China, the prevalence of cognitive impairment in people aged >65 years is the highest in the world, reaching 5.14% (Chan K. Y. et al., 2013). In 2010, the global medical expenses for cognitive impairment reached 6,040 billion US dollars (Wimo et al., 2010), representing an important health problem in this century.

Currently, there is no really satisfactory treatment for AD. AD is a progressive cognitive decline, with MCI being the predementia state. Elderly people with MCI have an increased probability of conversion to AD (Brooks and Loewenstein, 2010). Early intervention in MCI will be a key opportunity in the management of dementia (Levin et al., 2010).

A clinical guideline (Petersen et al., 2017) has indicated that there is no high-quality evidence to support that drug treatment can improve the clinical symptoms and delay the progression of MCI, and Food and Drug Administration (FDA) has not approved such drugs. The guideline has stated that regular exercise and cognitive function training can improve the cognitive function in MCI and clearly proposed that regular exercise twice a week can be used as the treatment of patients with MCI (level B recommendation) (Vidovich et al., 2009). Therefore, it is an imperative need to find new and efficient methods to treat MCI.

Jing et al. (2008) have shown that acupuncture could reduce the decline rate of cognitive ability in the elderly with MCI. Zhao et al. (2012) have found that acupuncture could significantly improve the learning and memory ability of MCI patients compared with nimodipine. A meta-analysis of 568 studies (Deng and Wang, 2016) has shown that acupuncture could significantly improve mini-mental state examination (MMSE) scores, considering that acupuncture can be used as an alternative treatment for patients with MCI. In the early stage, we performed the behavioral research of acupuncture at Baihui Point on rats with ischemic memory impairment, which proved that it could significantly improve the learning and memory ability of rats, and the mechanism was related to the upregulation of BDNF and TrkB expression (Lihong et al., 2018). Clinical studies on acupuncture for MCI/AD have also been performed. However, most of them were clinical studies, and there is still a lack of research on the effect mechanism of acupuncture in improving MCI from different dimensions.

The complexity and multifactorial etiology of AD pose unique challenges for studying its pathogenesis and developing therapies. Various pathological modifications occur some years before the first signs (Gauthier et al., 2018). The recognition of these biomarkers often requires invasive or radioactive testing and cannot be accepted when people have mild symptoms. Intestinal flora can affect the nervous system activity through the “gut-brain axis,” and increasing evidence has found that intestinal flora is also involved in the formation, processing, and storage of memory (Wang and Kasper, 2014; Alkasir et al., 2017). In view of the complex symbiotic relationship between the host and microorganisms, which can affect brain function through the bidirectional information exchange through the gut-brain axis, the gut is regarded as the “second brain” (Jiang et al., 2017). Recent studies have explored gut bacterial taxa and discovered their altered abundance in AD (Vogt et al., 2017; Zhuang et al., 2018).

The illumination of the gut-brain axis in MCI pathogenesis would require accessible biomarkers. The recent studies on MCI gut microbiota have revealed that microbiota was already altered before the onset of dementia (Li et al., 2019). The relationship between cognitive function and the microbial gut-brain axis is a very important field. However, the interaction between gut microbiota and acupuncture treatment has been scarcely investigated. Only one clinical study (Wang et al., 2022) have revealed that dysbiosis was found in the gut microbiome in subjective cognitive decline and partially relieved by acupuncture, Bifidobacterium is positively correlated with clinical efficacy Z-score and Montreal Cognitive Assessment (MoCA) Scale (both *P* < 0.005). Additionally, it is necessary to conduct systematic research on MCI elderly people to explore the prevention strategy of AD in the future, which can not only understand the evolution process of intestinal flora during and after acupuncture but also provide the basis for making the prevention plan for early cognitive impairment.

## Methods and analysis

### Objectives

The objective of our study is to evaluate the medium and long-term clinical efficacy and safety of acupuncture in subjects with MCI. Additionally, the study aims to investigate the changes in intestinal flora in patients with MCI before and after acupuncture treatment.

### Study design

**FIGURE 1:**
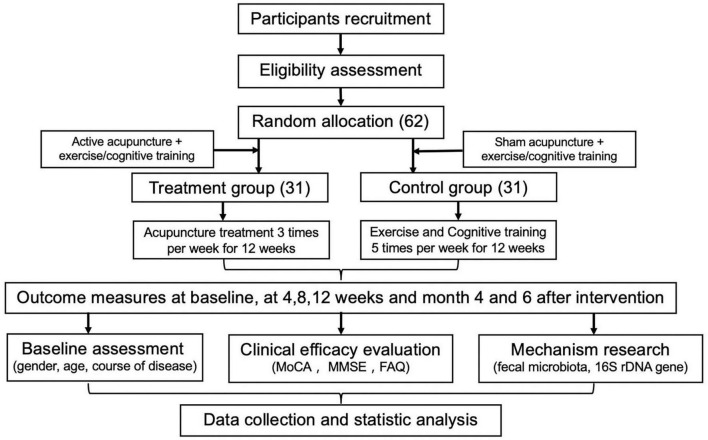
Study flow chart.

This study will be an open, single-center, randomized, assessor- and data analyst-blind controlled trial with a 1:1 allocation ratio. This protocol adheres to the Standard Protocol Items: Recommendations for Interventional Trials (SPIRIT) statement (Chan A. W. et al., 2013). The subjects (*n* = 62) will be randomly allocated to the treatment group (*n* = 31) and control group (*n* = 31). This project was approved by the Ethics Committee of the Zhejiang Hospital (2020-LS-11K) on 21 February 2020. Each patient will sign informed consent. The proposal is labeled in Figure 1.

### Study population, recruitment, and consent

Eligible patients aged 55–75 years with a diagnosis of MCI are recruited at Zhejiang Hospital.

Before inclusion, the study doctor will inform the patients by telephone. If the patient decides to join in the study, they will sign the informed consent form. Patient recruitment will be conducted between January 2021 and December 2022. Analysis of data, followed by the publication of the results, is expected in 2023.

### Inclusion criteria

(1) present cognitive impairment, which is confirmed by the informed person; (2) one or more damaged cognitive domains as determined by objective examination; (3) CDR score of 0.5; (4) normal activities of daily living score; (5) MMSE score of 24–28; GDS score of <9; (6) stable and reliable caregivers; (7) age of 55–75 years; (8) signed informed consent.

### Exclusion criteria

(1) Age of <55 or >75 years; (2) diagnosed with any type of dementia; (3) a history of acute or chronic cerebrovascular disease, encephalitis, Parkinson’s disease, Leukoencephalopathy, and other central nervous system diseases; (4) a history of hypothyroidism, vitamin B12 deficiency, depression, syphilis, and other diseases that may affect cognitive decline; (5) GDS score of ≥ 9 points; (6) severe hearing and visual impairment, inability to complete neuropsychological tests; (7) impaired liver and kidney function and hematopoietic system disorders; (8) the use of antibiotics and probiotics within the past month; (9) inflammatory gastrointestinal diseases, irritable bowel syndrome, chronic constipation, chronic diarrhea, *Helicobacter pylori* infection (untreated), and other diseases affecting the intestinal flora; (10) poor compliance and other conditions not suitable for acupuncture treatment.

The necessary number of participants will be enrolled at baseline.

### Sample size estimation

The study will be a single-center, randomized, controlled trial. The main outcome is whether cognitive function is relieved after acupuncture treatment. According to the preliminary experimental results, the rate of acupuncture treatment is about 90%, and the rate of cognitive function training is 67.9%. We found that the effective rate of our preliminary study is comparable with many previous studies (Wang and Luo, 2016; Sun et al., 2018; Yang et al., 2018). The sample size was calculated by using the following formula:


$$n=\frac{8\,p\,q}{{\left(p_{1-}\,p_{2}\right)}^{2}}$$

In the equation, N is the required sample size for each treatment group, and p1 and p2 represent the original curative effect and the expected curative effect of 67.9% and 90%, respectively. *p* = (*p*1 + *p*2)/2 =  0.79. *q* = (1-*p*1 + 1−*p*2)/2 =  0.21. By substituting the above-mentioned parameters and values into the formula and accounting for a 15% expulsion rate, the final estimated sample size is about 31 cases per group (62 in total).

### Interventions

Participants randomized to the treatment group will receive active acupuncture stimulation, exercise and cognitive training. The acupuncture will be operated by a senior acupuncturist. Traditional acupoints used will include EX-HN1 (Sishencong), GV20 (Baihui), GB13 (Benshen), GB20 (Fengchi), GB39 (Xuanzhong), ST36 (Zusanli), SI6 (Yanglao), and SP6 (Sanyinjiao). After skin disinfection in patient in the supine position, the adhesive pads were pasted on the acupoints surface. Then, the acupuncture needles were inserted through the adhesive pads approximately 1–3 cm into the skin with manipulation. During the procedure, the needle is kept for 30 min, and small-amplitude uniform lifting is given, inserting and twisting three times every 10 min.

Participants randomized to the control group will receive sham acupuncture stimulation, exercise and cognitive training. Sham acupoints (1 cm away from the acupoint) that are separate from conventional acupoints or meridians were used for the control group. Patients received non-insertive acupuncture using the pragmatic placebo needles. As same as treatment group, the adhesive pads were firstly pasted on the sham acupoints surface. The pragmatic placebo needles with a blunt tip were placed on the adhesive pads. In order to minimize the physiological effect, acupuncturists were instructed to lightly place the placebo needle on the adhesive pads with no manipulation.

Active or sham acupuncture treatment sessions will be performed daily for 3 days per week for 12 weeks.

The exercise and cognitive training measures will be analogous to those of the control group. Exercise training will include Tai Chi, square dance, ball games, aerobic gymnastics, jogging, and other aerobic exercises acceptable to patients (3 times a week, 1 h/time). Cognitive training will comprise the following. (1) Attention training in which the differences are found by the patients between the two images, materials, and words, gradually strengthening the attention span and continuity of the patients. (2) Memory training in which the patient will gradually recall what happened in the past through previous pictures and materials and will be provided with appropriate reminders to enhance the patient’s confidence. (3) Understanding and judgment training in which patients will participate in chatting, playing chess, playing cards, and other activities every day to help patients improve their understanding and judgment. (4) Logical thinking ability in which patients will be encouraged to participate in family economic management and family affairs decision-making to integrate them into the family and society. (5) Communication training in which patients will be encouraged to read aloud, communicate with relatives and friends around them, and actively express themselves. The first three times of cognitive training will be trained by professional rehabilitation doctors and rehabilitation therapists, and family members will be trained at the same time. The subsequent training will be completed by family members at home. At the 4th and 8th weeks, the rehabilitation doctors will decide whether to adjust the training program after assessment. Training 5 times/week for 1 h/time, emphasizing the patient as the main body, and the time and intensity will follow the principle of individualization. Continuous treatment will be provided for 12 weeks.

In the study, patients will be advised not to accept other treatment methods. If other treatment methods are needed, they will need to inform the doctor and record them.

### Randomization

Participants will be randomly divided into treatment (active acupuncture + exercise/cognitive training) and control groups (sham acupuncture + exercise/cognitive function training) using the method of a random parallel controlled trial. Qualified subjects will be numbered and grouped by the random grouping numbers generated by SPSS software to ensure the balance between the two groups.

### Blinding

We will try to ensure that participants accept comparable treatment measures. All researchers and statisticians responsible for data recording will be blind to random states.

### Clinical assessment

The recruited subjects will be evaluated by a neurologist. Clinical data will be collected through direct inquiry with participants and systematic evaluation of past medical histories, including the age of onset, history of cognition impairment, type of onset, number of episodes, family history, and drug use history. Additionally, we will collect detailed information about defecation status, the number of completely autonomous bowel movements per day, and stool characteristics (Bristol stool form scale, BSFS). Since this is related to microbiota analysis, data on patients’ diets will also be gathered by the food frequency questionnaire (FFQ) (Balasubramanian et al., 2020; Gutierrez et al., 2021). Psychobehavioral symptoms are a risk factor for the conversion of MCI to dementia, and even mild psychobehavioral symptoms increase the risk of conversion from MCI to dementia or AD (level I evidence) (Professional Committee of cognitive disorders, and Chinese Medical Association., 2018 Chinese guidelines for diagnosis and treatment of dementia and cognitive impairment (V): diagnosis and treatment of mild cognitive impairment 2018). The guidelines have indicated that attention and evaluation should be paid to the mental behavior of patients with MCI. The GDS created by Brank et al. is specifically used for the screening of depression in the elderly (Chan, 1996) and will be used in this study.

### Outcome measurements

Scales of clinical dementia rating (CDR, MMSE, MoCA) and activity of daily living (ADL) will be collected at baseline, during the treatment (weeks 4 and 8), at the end of the treatment, and at months 4 and 6 after the intervention.

### Primary outcome

According to the research objectives, the improvement of cognitive function assessed by MoCA will be regarded as the main result of the study. MOCA covers a wide range of cognitive fields, including attention and concentration, executive function, memory, language, visuospatial structure skills, abstract thinking, calculation, and orientation. It is specifically designed to screen for MCI with high sensitivity (80–100%) and purposefulness (50–76%) (Nasreddine et al., 2005).

The patient’s feces will be stored in the refrigerator at -80°C. 16S rDNA sequencing will be used to detect the changes in intestinal flora distribution in patients.

### Secondary outcomes

Secondary measures will include the MMSE and the functional activity questionnaire (FAQ) scores.

MMSE has 11 questions and tests five aspects of cognitive function. The highest score is 30 points, and a score of <24 points is regarded as abnormal dementia screening (Vidovich et al., 2009).

Daily abilities include basic activities of daily living (BADL) and instrumental activities of daily living (IADL). The former refers to the most basic abilities required for independent living, and the latter refers to the ability of complex daily or social activities. The diagnosis of MCI requires normal BADL and slight impairment of IADL. FAQ is one of the commonly used IADL evaluation scales, which has been proved to distinguish between people with normal cognitive function and those with MCI (level II evidence) (Professional Committee of cognitive disorders, and Chinese Medical Association., 2018 Chinese guidelines for diagnosis and treatment of dementia and cognitive impairment (V): diagnosis and treatment of mild cognitive impairment 2018) and can be used as an indicator of the transformation from MCI to AD (level II evidence) (Hsiao et al., 2015).

### Incidence of adverse events

Any adverse events occurring during the treatment will be recorded, including occurrence time, degree of performance (mild, moderate, and severe), treatment process, correlation with treatment (definitely related, possibly related, possibly unrelated, irrelevant, and unable to judge), and prognosis. In the case of serious adverse events (fatal, life-threatening, requiring hospitalization, prolonging hospitalization, causing permanent or temporary organ dysfunction, etc.), the treatment will be stopped immediately regardless of whether it is related to treatment or not.

### Analysis method of fecal microbiota

With the development of next-generation sequencing high-throughput sequencing technology, researchers can obtain huge data information based on the 16S rDNA gene and second-generation sequencing technology. The diversity of microorganisms in each ecological community will be analyzed by bioinformatics. Statistical analysis will be used to study whether there are differences in the main components of microorganisms in different ecological communities. Further, the composition of microbial communities related to environmental factors, such as diseases, will be evaluated through ribosomal database project (RDA) analysis to clarify the interaction relationship between microorganisms and the environment (host) and the adaptive mechanism of the microbial environment (host environment) (Klindworth et al., 2012).

### Data analysis plan

V3/V4 sequence will be a proved error correction protocol. Quality filtering, including pruning low-quality bases at the beginning and end of each reading with a sickle,^1^ before assembling paired readings with PEAR (V.0.9.1) or equivalent products and Bayes Hammer will be used for error correction (Nikolenko et al., 2013; Zhang et al., 2014). The size selection filter and BLAST search will be set to the expected amplifier size to ensure that PHiX spikes in control are detected and removed.

QIIME (Caporaso et al., 2010) will be used to analyze these corrected and assembled readings. USEARCH will be run using *ab initio* and open reference operation classification units (OTU) clustering methods and *ab initio* chimerism detection using UCHIME V.5.1 (Edgar, 2010; Edgar et al., 2011). Classification will be assigned to operational taxonomic units (OTUs) by naive Bayesian RDP classifier using Silva and GREENGENES classification database.

The Student’s *t*-test will be used to compare the estimates of species richness (OTUs number) and diversity (Shannon index) in the sample of MCI patients at multiple sparse depths. Weighted and unweighted UniFrac distances will be used to assess potential clustering of microbiota composition, as well as the degree of overlap in composition. These results will be visualized using principal coordinates analyses, and statistically significant clusters will be identified using Adonis. Random forest will be used to identify OTUs that distinguish individuals.

Based on the linear discriminant analysis effect size method (LefSe), we will compare the genera between the MCI patients from fecal samples, respectively. Considering the difference in the genus classification group, the score of log linear discriminant analysis is >2.0, and the *P*-value is set at <0.01.

### Data analysis

Using spss21.0 statistical software, the measurement data are expressed as mean ± standard deviation. The counting data are expressed by frequency. Pearson’s χ^2^-test or Fisher’s exact test will be used to analyze classified variables. The *t*-test will be used if the normal distribution and homogeneity test are satisfied, and the rank sum test will be used for non-normality or non-uniformity of variance. Kruskal Wallis test will be used for multigroup comparison, and the Wilcoxon rank sum test will be used for comparison between the two samples. For repeated measurement data, repeated measurement analysis of variance will be used. For the pairwise comparison of multiple groups of data, the Bonferroni method will be used to adjust the test level.

## Discussion

MCI is considered a dangerous state of possible further cognitive impairment. Now, there is no available drug to prevent cognitive deterioration in patients with MCI. The purpose of this study is to estimate whether acupuncture can improve cognitive function in patients with MCI and explore the relationship between acupuncture mechanism and changes in the intestinal flora.

Currently, the research on the intestinal microbiota has found that the intestinal microbiota changes before the onset of AD. However, the relationship between gut microbiota and treatment type is still not known. Therefore, in this study, we seek to explore this relationship. We hope to be able to provide assessable biomarkers associated with acupuncture treatment.

We adopted the acupuncture treatment procedures most commonly used in previous clinical trials on cognitive impairment and dementia (Lee et al., 2012; Cao et al., 2013; Deng and Wang, 2016). The rationale for choosing the acupoints is as follows. The acupoints on the head, such as EX-HN1 (Sishencong), GV20 (Baihui), GB13 (Benshen), and GB20 (Fengchi), have the local effect of regulating brain function. The other acupoints on the limbs, such as GB39 (Xuanzhong), ST36 (Zusanli), SI (Yanglao), and SP6 (Sanyinjiao), have the effect of tonifying the kidney and spleen, regulating the function of the stomach, and promoting the production, operation, and distribution of Qi, blood, essence, and fluid to fill the marrow sea and nourish the brain (Yang et al., 2003). Functional magnetic resonance imaging has been widely used to investigate the effects of acupuncture on neural activity. The acupoints can regulate brain networks by increasing connectivity between cognition-related regions, thereby improving cognitive function in patients with MCI (Tan et al., 2017). By improving brain function, acupoints further affect the movement, sensation, and secretion of the gastrointestinal tract through the descending pathway of the “intestinal brain axis” and regulate the composition and behavior of intestinal microorganisms (Furness, 2012). Clarification of the relationship between acupoints and intestinal flora is the objective of our study.

During the trial, effective blinding will support keeping comparability between groups. All researchers and statisticians who record the measurement data will be blind to the random state. The allocation concealment method seems to fully prevent possible bias.

Before beginning the trial, a colleague who will not participate in the baseline evaluation will be responsible for preparing sequentially numbered, opaque, and sealed envelopes containing intervention tasks according to the random list generated by the computer program. After patients participate in the trial, the acupuncturist will open the envelope to find the intervention task.

Most acupuncturists perform personalized interventions according to patients’ symptoms and traditional Chinese medicine (TCM) diagnosis. Considering that the formulaic and fixed acupuncture scheme is easier to be popularized worldwide and reduce the research deviation, we chose a unified acupoint for all participants in the study.

We acknowledge the importance to the vascular contributions to cognitive function (Custodero et al., 2022; Kiss et al., 2022; Montagne et al., 2022). Acupuncture was found can alleviate cognitive function in Vascular dementia (VD), but the underlying mechanisms are still unclear (Cao et al., 2013). Research showed that acupuncture was notably repressed the expression of inflammatory cytokines in the hippocampus and plasma of VD rats. The researchers proposed acupuncture attenuates cognitive impairment associated with inflammation through inhibition of the miR-93-mediated TLR4/MyD88/NF-κB signaling pathway in experimental VD, and acupuncture may be an underlying TLR4 inhibitor for the treatment of VD (Bu et al., 2022). Regarding the pathway for pathogenetic factors to the brain, Braniste et al. (2014) found the relationship between the lack of normal gut flora in germ-free mice and increased BBB permeability in adult and embryonic animals. The disruption or absence of the microbiota in mice impaired the function of the BBB and induced abnormal central nerve system (CNS) function, including cortical myelination, hippocampal neurogenesis, cognitive function, and memory formation (Sharon et al., 2016). In this way, systemic inflammatory reactions caused by compounds secreted by bacteria may impair the BBB and promote neurodegeneration (Dinan and Cryan, 2016; Francesca et al., 2016; Quigley, 2017). We assumed that specific microbiota clusters may contribute to breakdown of the BBB. This could be important for our future research.

The protocol has some limitations. First, because of the lack of adequate preliminary studies, the study was designed as a single-center pilot study. Second, because of the limited research fund and lack of adequate eligible patients, the sample size is small.

At the end of this research, the prevention potential will be discussed based on the obtained results. The research results are expected to provide evidence for the efficacy of acupuncture in improving the cognitive function of MCI patients.

## Conclusion

Acupuncture is an effective method to improve cognitive function in MCI. This study will provide data on the relationship between gut microbiota and the effects of acupuncture in patients with MCI from a new angle.

## Data availability statement

The original contributions presented in this study are included in the article/supplementary material, further inquiries can be directed to the corresponding author.

## Ethics statement

The studies involving human participants were reviewed and approved by Zhejiang Hospital (2020-LS-11K). The patients/participants provided their written informed consent to participate in this study.

## Author contributions

YJ and JZ conceived the study, drafted the original protocol, and provided critical revision of the final draft. YJ provided statistical expertise. FH supplied information about recruitment, consenting, clinical management, finances, record keeping, participant withdrawal, and reporting. JZ provided information and standard operating procedures on microbiota methods and immunoassays. All authors involved in the revision of the protocol manuscript, agreed to the final content, and were accountable for the accuracy and integrity of the work.

## Footnotes

1
https://github.com/najoshi/sickle

## Abbreviations

MCI: Mild cognitive impairment
MMSE: mini-mental state examination
MoCA: Montreal Cognitive Assessment
AD: Alzheimer’s disease
CDR: clinical dementia rating
GDS: Geriatric Depression Scale
FDA: Food and Drug Administration
ADL: activity of daily living
FFQ: food frequently questionnaires
FAQ: functional activity questionnaire
OUT: operational taxonomic units
CNS: central nerve system
rRNA: ribosomal RNA.
